# P-328. Compliance of Hand Hygiene Practice among Health Care Workers in Tertiary Care Military Hospitals of Bangladesh

**DOI:** 10.1093/ofid/ofae631.531

**Published:** 2025-01-29

**Authors:** Syed Abul Hassan Md Abdullah, Md Golam Dostogir Harun, Md Shariful Amin Sumon, Ishrat Jahan, Md Mahabub Ul Anwar, Taufiq H Siddiquee

**Affiliations:** Bangladesh University of Professionals, Dhaka, Dhaka, Bangladesh; icddrb, Dhaka, Dhaka, Bangladesh; icddr,b, Dhaka, Dhaka, Bangladesh; Army HQ, Bangladesh Army, Dhaka, Dhaka, Bangladesh; Office of Health Affairs, West Virginia University, USA, Dhaka, Dhaka, Bangladesh; BRAC Healthcare, Dhaka, Dhaka, Bangladesh

## Abstract

**Background:**

Hospital-acquired infection is still a major cause of morbidity and mortality in hospitals. Healthcare workers’ (HCWs) hands become progressively colonized with potential pathogens during their patient care and act as a vehicle for transmission of microorganisms to other patients. Hand hygiene (HH) is undisputedly one of the most effective infection control measures. Military hospitals in Bangladesh are known for better compliance of health system practice. This study aimed to evaluate HH compliance and associated factors among HCWs in two tertiary level military hospitals in Bangladesh.

**Table 1:**
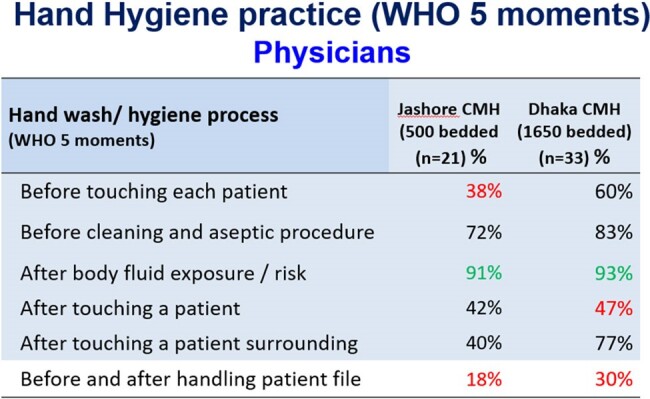
HH practice of Physicans

**Methods:**

We conducted non-participatory observations at 02 tertiary Combined Military hospitals (CMH) at Dhaka (1650 bedded) and Jashore (500 bedded) during Oct to Dec 2021. We used the WHO’s ‘5 moments of Hand Hygiene tools’ to record HH compliance among physicians, nurses and cleaning staff. We also performed semi-structured interviews to determine the key barriers to complying with HH.

**Table 2:**
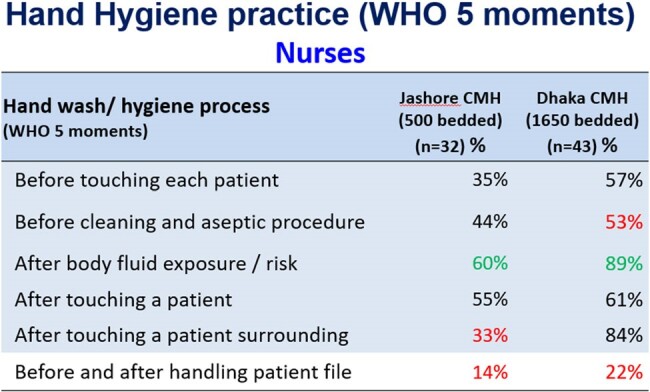
HH practice of Nurses

**Results:**

We observed total 172 HH opportunities. The overall HH compliance was 30.9%, highest among physicians (34.5%), followed by Nurses (25.2%). Physicians’ performance (49%) on HH practice before touching a patient was higher than the nurses (46%) but poorer (44.5%) after touching the patient than same practice of nurses (58%). Study also observed that only 24% physicians and 18% nurses practiced HH Before and after handling patient file. Responded opined that workload, shortage of time, lack of facility, distance of hand washing station, inadequate supply were the key hand hygiene barriers with soap. Insufficient supply, skin reaction, cost of product, lack of knowledge appeared. Qualitative assessment revealed that Inadequate handwashing station at wards (distance), insufficient training & orientation for HCWs, irregular monitoring and audit and lack of enforcement of IPC guidelines were key challenges at both study sites.

**Table 3:**
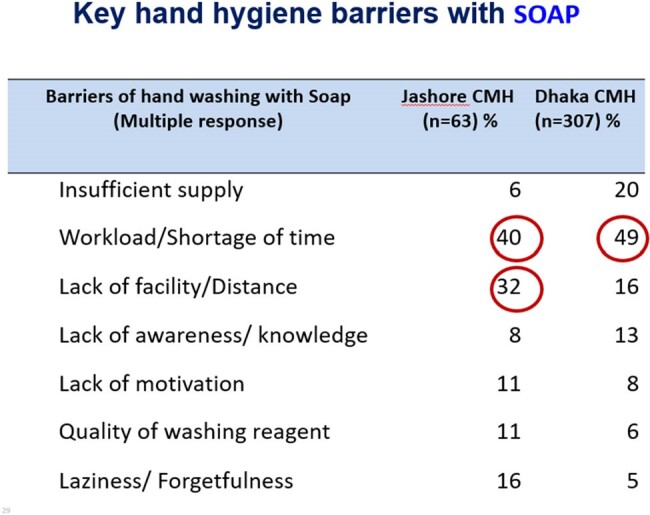
HH practice barrier with soap

**Conclusion:**

HH compliance among HCWs of study hospital was short of standard for safe patient care. Periodical training and motivation, ensuring HH supplies and enforcement of IPC guideline is needed to improve the situation.

**Table 4:**
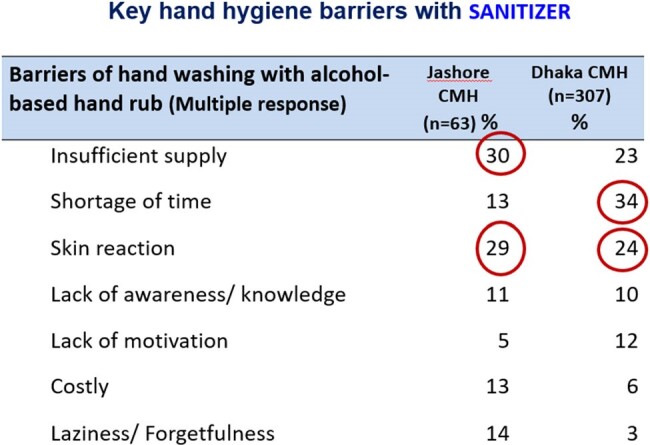
HH practice barrier of sanitizer

**Disclosures:**

**All Authors**: No reported disclosures

